# Evidence-based art in the hospital

**DOI:** 10.1007/s10354-021-00861-7

**Published:** 2021-08-02

**Authors:** Axel Fudickar, Dag Konetzka, Stine Maria Louring Nielsen, Kathy Hathorn

**Affiliations:** 1 Department of Anaesthesiology and Intensive Care Medicine, Christian-Albrechts-University Kiel, University Hospital Schleswig-Holstein, Campus Kiel, Arnold-Heller-Str. 3/R3, 24105 Kiel, Germany; 2 Department of Architecture Design & Media Technology, Aalborg University Copenhagen, Copenhagen, Denmark; 3 American Art Resources, Houston/Texas, USA

**Keywords:** Art effects, Figurative art, Biophilia, Isolation, Abstract art

## Abstract

**Background:**

Evidence-based art is the investigation of art effects and art investigated for effects. In this study the evidence regarding patient preferences for art styles and effects of art in nonpsychiatric hospitals and outpatient departments was reviewed.

**Methods:**

Results from original articles were retrieved by a scoping PubMed search and by browsing the internet using the terms “evidence based art”, “evidence based design”, “art and hospital” and “design and hospital”, “art effect”, “design effect”, “landscape preference” and “abstract art figurative art”. The quality of art was not operationalized as a criterion.

**Results:**

Of the articles 7 original sources showed patient preference for natural scenes and figurative art, 2 studies showed no preference, 16 studies showed positive art effects on well-being and behavior and 5 studies showed a positive effect of nature pictures on measurable findings.

**Conclusion:**

Controversial results together with theoretical aspects suggest natural scenes in patient rooms and diverse art in public areas.

## Background

Quick worldwide dissemination of SARS-CoV‑2 (severe acute respiratory syndrome coronavirus type 2) leads to an increasing number of isolated and lonely patients and a part of them dies in this situation. Isolation due to any disease in a boring surrounding exposes vulnerable subjects to psychological stress and pathological symptoms. It is common sense that such situations can be improved by visual artwork like pictures or videos. A survey in Utah revealed that art in patient rooms was more important for patients than hospital architecture, surrounding, social facilities or parking space [1]; however, usually not patients, but clinic staff, artists or art committees decide what kind of art is placed in patient rooms. From a sociological point of view this may fail to meet patient’s needs, because patients with different social backgrounds may have problems to understand the artwork in a way that was intended by those who decide about art in hospitals [2, 3].

The research topic “Evidence-based art (EBA)” as a part of the topic “Evidence-based design” aims at the objective measurement of effects of art on well-being and pathological symptoms [4, 5]. The EBA should provide empirically founded recommendations, how art can be used in hospitals with benefit for patients [6]. Some early studies resulted in positive effects on well-being, length of hospital stay, stress, pain and analgetic consumption, so-called minor complications, depressions, anxiety and mood [7].

Aim of this scoping review is to summarize the results of original studies regarding art preferences and effects of art in nonpsychiatric hospitals and to derive recommendations for practice considering theoretical aspects and experiences of hospital design.

## Methods

Literature search for this scoping review was based on a guideline to EBA by Hathorn and Nanda and performed by a scoping search in PubMed and an internet search using the key words “Evidence Based Art”, “Evidence Based Design”, “art and hospital”, “design and hospital”, “art effect”, “design effect”, “landscape preference” and “abstract art figurative art” [8]. Sources published before May 2021 were included.

## Results

Literature search resulted in 30 original sources investigating preference for art styles and effects of visual art in nonpsychiatric hospitals. The articles were divided in three groups including preferences for certain art styles, effects on wellbeing and behavior and effects on findings (Table 1).

**Table 1:** Evidence based art studies in hospitals

Endpoint	Reference	Method	*N*	Result
Art Preference	Carpman 1993 [9]	Interview	300	Natural views are preferred against other motives
	Hanson 2013 [10]	Questionnaire	80	Preference for natural views
	Frandsen 2014 [11]	Interview	100	Patients prefer natural views
	Nielsen 2017 [12]	Interview	103	Patients prefer figurative art
	Nanda 2008 [13]	Interview	67	Patients prefer natural views over abstract art even if potential bias for quality is considered
	Nanda 2012 [14]	*Interview*	144	Patients prefer natural views over abstract art even if natural views are compared with best-selling popular abstract art
	Nanda 2009 [15]	Rating scale	64	Children of all age groups preferred bright and colorful nature views with strong context
	Staricoff 2001 [16]	Interview	91	Preference of art is independent of art style (Landscape vs. abstract)
	Tovborg 2019 [17]	Interview	600	Art preference of patients and personnel is variable and situation-dependent
Effects on well-being and behavior	Diette 2003 [18]	Intervention	108	Pain reduction by natural views during bronchoscopy
	Miller 1992 [19]	Intervention	17	Reduction of pain and fear by natural views in burn patients
	Schneider 2003 [20]	Intervention	16	Virtual art and nature scenarios reduce fear during chemotherapy
	Tse 2002 [21]	Intervention	46	Nature views increase pain threshold and pain tolerance in probands
	Frandsen 2014 [11]	Interview	100	Positive effect of art on mood and well-being
	Suter 2007 [22]	Interview	37	Improvement of mood by own choice of pictures
	Staricoff 2001 [16]	Interview	91	Positive effect of art on mood and stress independent of art style (landscape vs. abstract)
	Bonett 2015 [23]	Interview	46	Natural views at the ceiling estimated as positive by patients during radiation therapy
	Nanda 2012 [24]	Observation Noise level measurement	n.a.	Positive effect of natural views on behavior of waiting patients and noise level in an emergency department
	Nielsen 2017 [12, 25]	Interview Thermal camera	68	Art increases well-being independently of art styles Art in day rooms increases patient interaction
	Moss 2013 [26]	Interview	20	Art promotes feeling of care, socialization and finding of new interests
	Nielsen 2017 [27]	Multidimensional anthropological evaluation EEG, Eye-Tracking	30	Abstract and figurative art can have a positive effect on patients Reception of abstract art induces less cognitive strain than reception of figurative art
	Karnic 2014 [28]	Questionnaire	1094	Positive effect of a clinical modern art program on mood, stress, comfort, and expectations
	Nanda 2002 [29]	Questionnaire	210	Hospital art makes patients and visitors feel better
	McCabe 2013 [30]	Interview/Scale	199	Effect of visual art on levels of anxiety and depression
Effects on findings	Heerwagen 1990 [31]	Measurement of heart frequency	40	Pictures in the waiting area reduce heart frequency and restlessness
	Coss 1996 [32] [32]	Measurement of blood pressure	72	Nature views reduce preoperative blood pressure
	Harper 2015 [33]	Measurement of blood pressure	117	Natural views in examination rooms reduce blood pressure
	Ulrich 1993 [34]	Measurement of analgetic consumption	166	Natural views reduce analgetic consumption
	Ulrich 2003 [35]	Measurement of pulse-rate and blood pressure	591	Pulse-rate was lower in a nature environment design versus an urban environment design

Summary of included evidence based art studies in hospitals

*N* case number, *n.a.* not applicable

### Preference for art styles

An investigation of patient preferences for art styles at the University of Michigan Medical Center in Ann Arbor resulted in a recommendation for texturally complex nature views as artwork in patients’ rooms, but not for urban views, portraits, architecture, still life, sports, abstract art or poster art with text. It was also discouraged from muted or dark colors [9]. In another study photographs were displayed to 80 cancer patients. Most preferred motives were lake sunset (76%), rocky river (66%), and autumn waterfall (66%). Most disliked motives were park (54%), farmer’s market vegetable table (51%), and kayakers (49%). Preference of categories were landscape (28%), animals (15%), people (14%), entertainment (10%), imagery (10%), water (7%), spiritual (7%), flowers (6%), and landmark (3%). However, water was also depicted in some landscape motives. Thus, the ranking for water may be biased [10]. In a further study, patients, who were requested to choose the pictures in their rooms, also choose nature views in most cases [11]. This preference for nature views and figurative art was confirmed as one result of a study about patient behavior in dayrooms [12]. In addition to this, preference for natural views over abstract art prevails even if bias for quality is considered and natural views are compared with best-selling popular abstract art [13, 14]. Preference for natural views was also confirmed in a study including 64 children. Children of all age groups preferred bright and colorful nature views with strong context [15]; however, a study at the Chelsea and Westminster Hospital in England showed no preference for a certain art style, when nature scenes and abstract works were displayed on patient wards [16]. Concordantly, the Danish sociologist Anette Stenslund found very variable and situation dependent preference for different art styles when interviewing patients and staff in a hospital [17].

### Effects on well-being and behavior

Effects of art on well-being and behavior have been observed in various settings. Nature views at the ceiling reduced pain during flexible bronchoscopy [18]. Wall paintings reduced pain and anxiety in patients with burns [19]. During chemotherapy anxiety and fatigue were reduced by underwater scenes presented as virtual reality [20]. A Chinese study showed that nature videos can increase pain threshold and pain tolerance threshold [21]. The opportunity to choose the artwork in the patient’s room had a positive influence on well-being, mood and feeling of self-control in further investigations [11, 22]. A positive effect on wellbeing has also been described for fixing of nature views above a computed tomography [23]. Moreover, it could be shown that nature views in emergency departments can result in positive modification of patient behavior [24]. In addition to this, a combination of ethnographic observations and thermal camera recordings showed that both figurative and abstract art can influence well-being. This was explained by improved environment, enhanced social interaction, increased self-esteem and a virtual connection to the activities outside the hospital [25]. This has been confirmed in a qualitative study including 20 older patients [26]. A further study investigating six dimensions of well-being with ethnographic interviews and psychological experiments (EEG and eye tracking) showed that abstract art can also have positive effects on wellbeing. Independently of the style, art leads to more frequent interactions in dayrooms and a higher rate of contentedness in patients with different somatic diseases [27]. A questionnaire-based evaluation of a comprehensive modern art program at Cleveland Clinic, USA including 1094 patients resulted in a significant effect of the art program on mood, stress, comfort, and expectations [28]. In a further study 210 patients or visitors rated the effect of art in well-being in a clinic overall, in the waiting room and in the examination and consultation rooms while waiting by filling a 2-page survey. 84% of patients or visitors agreed that the artwork made them feel much better (15%) or better (68.5%) [29]. This was confirmed by a study showing an effect of visual art on levels of anxiety and depression in cancer patients [30]*.*

### Effects on findings

Measurable findings were also positively influenced by art in hospitals. In a dental clinic stress in the waiting area, measured by heart frequency and questionnaires was reduced in 40 patients by a huge wall painting [31]. Ceiling pictures with calm nature views resulted in lower mean blood pressure in 72 patients waiting for surgery [32]. This effect was reproduced in 117 patients in examination rooms [33]*.* A study from Sweden resulted in less consumption of strong analgetics and less anxiety after heart surgery in intensive care patients exposed to nature views after heart surgery. Abstract art led to more anxiety than no pictures in this investigation [34]. Pulse-rate was lower in a nature environment design versus an urban environment design in 591 study participants in a further study [35]*.*

## Discussion

Patients preferred figurative art and nature views in many, but not all studies and most studies resulted in a positive effect of figurative art including nature views on wellbeing and findings. Based on these results theories explaining this preference were outlined by some authors.

### Possible explanations for a preference for figurative art

According to emotional congruence theory human beings scan the environment for patterns corresponding to their emotional state. Hence, following this theory abstract pictures with negative or undefined contents could trigger or enhance negative emotions in viewers under difficult life conditions [6, 35, 36]. Pleasant figurative pictures could provide a positive distraction to harmless stimuli [37]. These interpretations are confirmed by the fact that figurative pictures have a higher concordance of aesthetic preference between different viewers than abstract pictures [38]; however, figurative art can also be more sinister and aggressive than abstract art, if inappropriate motives are depicted.

### Explanations for a preference for natural views

According to the theory of psychological evolution human beings experience nature views as calming and refreshing due to their adaptation to survival in nature, because a tendency to fecund and watery areas (biophilia) may have anchored a preference for the corresponding pictures and colors in human brains [7]. Consequently, everything reminding of dangerous situations like patterns associated with hostile animals and poisonous plants or aggressive faces has to be avoided according to this hypothesis [35]. Patients recovering after cholecystectomy in a room with a window with view on a natural scene recovered quicker than patients with view on a brick wall in a study [39]. Biophilia is confirmed by an older study resulting in less analgesic consumption, less minor complications and earlier discharge from hospital of patients with a view on nature in comparison with patients who had a view on a wall [40].

Moreover, nature views can distract from stressful events, because they bind awareness by positive associations including the notion of being far away, fascination by spectacular sights, feelings of freedom and connection with the outer world [41]. Aesthetic experience of nature views is also easier for patients with cognitive impairment by stressful events, because nature views are easy to understand due to their familiarity [41].

### Recommendations for nature views in hospitals

**Fig. 1:**
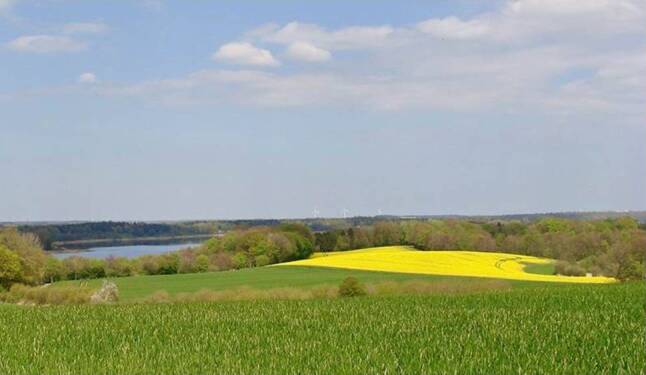
Landscape photo with picture elements estimated as positive by patients: Open familiar landscape with calm water and shade-giving trees as relaxing diversion from clinical routine (Susanne Kollmann: “Landscape”, Muthesius University of Fine Arts and Design, Kiel). Abdruck mit freundlicher Genehmigung. Diese Abbildung fällt nicht unter die Creative Commons CC BY-Lizenz dieser Publikation

Based on these results and theoretical considerations, recommendations for contents of art in hospitals have been proposed. Preferred motives include open, savannah-like landscapes with calm water and umbrella-like trees, beautiful old buildings, gardens with flowers or friendly faces (Fig. 1; [6, 35]). In addition, art location in the hospital should be taken into consideration regarding visibility in treatment rooms as well as special patient’s needs depending on disease, length of stay, prognosis, age, cultural background and sex, albeit criteria for these subgroups have not yet been elaborated [8].

### Art in isolation

The special problem of isolated patients without allowance to receive visits is roughly comparable to similar situations in space stations, jails, U‑boats or research bases in remote areas. The National Aeronautics and Space Administration (NASA) sponsored a study on the effect of natural views on blood pressure and stress parameter. In this study a positive effect on these parameters was correlated with experienced deepness of the depicted landscapes. Participants preferred natural views and contrary to the notion that the size of a picture may be decisive for its effect, small formats were also effective [42]. These results were confirmed by a further NASA study investigating 320 students, university faculty, and staff. The participants preferred nature views with wide open spaces and these nature views reduced physical arousal measured by pupillary dilation [43]*. *Negative effects were shown for close views of wild animals staring at the viewer [44]. Those effects could also be applicable for isolated patients (and personal caring for them).

### Biophilia in studies on non-hospitalized persons

The theory of biophilia in hospitalized patients has been supported by studies revealing biophilia in non-hospitalized adults and children. A preference for savannah landscape was also shown in 80 African teenagers and adult students who lived in landscapes different from savannah [45]. In part contrary to the savannah hypothesis, a comparison of nature view preferences between six different biomes in 274 college students revealed that tundra and coniferous forests were the most preferred landscapes [46]. In a study comparing preference for 4 different outdoor views in 105 5‑year-old children, the participants preferred parks with water not only as part of an attractive playground but also aesthetically [47]. These findings confirmed older recommendations for the integration of water areas into outdoor play environments [48]. A further study was specially focused on the effect of water in pictures showing in 68 adults that water views were preferred independent of whether additional picture elements contained natural or built environments [49]. A meta-analysis on 32 studies resulted in an increase of positive affect by exposure to natural environments albeit with heterogeneity depending on the type of emotion assessment, type of exposure to nature, location of study, and mean age of sample [50]. A more effective stress-reducing effect has been shown for nature posters in comparison to mixed and abstract posters by investigation of 210 student reactions to stress provocation in different office designs [51]. Hence, the preference for landscapes is not limited to the confined situation of hospitalized patients or similar isolated situations but seems to be an evolutionary developed characteristic of man [45]*.*

### Abstract art in hospitals

**Fig. 2:**
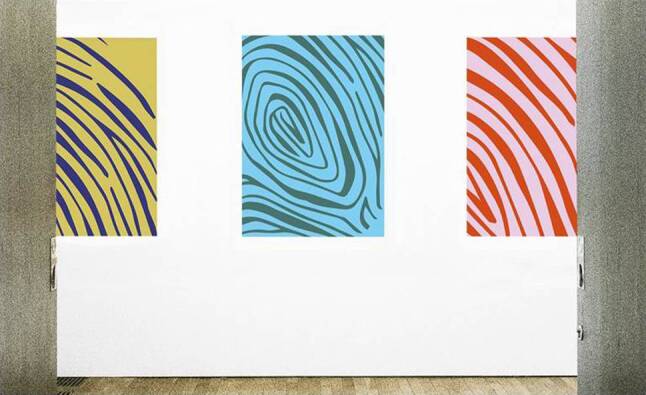
Abstract art intended for use in hospitals with mood-boosting colors. Abstract forms inspired by fingerprints associate changing individuality and helping hands and motivate reflexion (Anja Germanova: “Fingerprints Raumansicht”, Muthesius University of Fine Arts and Design, Kiel). Abdruck mit freundlicher Genehmigung. Diese Abbildung fällt nicht unter die Creative Commons CC BY-Lizenz dieser Publikation

The comparably large number of studies performed with nature views and figurative art may bias the interpretation of all studies considering EBA towards an overestimation of the effect of natural views. In spite of many arguments and study results favoring figurative and natural art in hospitals no preference for nature views or figurative art has been found in two studies [16, 17]. These studies support the notion of a variable art preference corresponding to the very different art preference in daily life. Moreover, two studies by Nielsen et al. showed by anthropological methods that abstract art can have positive effects on patient well-being [25, 27]. An advantage of abstract art over figurative art is the huge number of possible interpretations meeting various cognitive needs of different viewers [12]. Moreover, the confrontation with abstract art should enhance the capability to manage the consequences of disease for daily life by enabling a new perspective on the situation [52]. In this context, art is seen as a means to consider patient’s subjective situation according to the World Health Organization’s new definition of disease and health in connection with the cultural background [53]. Thus, not only the need for a pleasant environment with references to nature and daily life, but also the human need for stimulating sensual experiences is met by abstract art (Fig. 2; [52]).

Abstract art can also enable an emphasis on color effects on patient well-being. A preference of light colors and a greater importance of color and brightness for patient preferences has been suspected [25]. Although there is not much scientific evidence available, some interesting art projects in hospitals, e.g. the design of the Danish hospital in Herlev by Poul Gernes focus on a positive effect of colors [54].

Arousing curiosity as desire for new knowledge or avoidance of boredom and sensory deprivation has also been proposed as an important means to improve well-being by abstract art. Following incongruity theory, curiosity will be raised, if perceptions do not follow the rules of our common worldview, e.g. by novelty, surprise incongruity or complexity. Curiosity motivates exploration that is rewarded especially for new situations with moderate level of novelty including familiar as well as new information. This positive explorative behavior can distract from stressful events and enhance self-control. Moreover, to explore physical and psychic phenomena and combine new experiences to a defined spiritual worldview can help to master stressful situations [55]*.*

## Limitations

Results from studies regarding EBA are not regularly considered if art programs for hospital projects are designed [4]. An argument against unlimited application of EBA is the comparably low evidence grade of available studies. Power analyses, exact definition of clinical endpoints and defined statistical analysis are frequently lacking and some studies are qualitative studies or anthropologic case collections. Study design, availability of primary sources and completeness of reports are very inhomogeneous. Hence, no systematic quality assessment or quantitative analysis of the identified studies could be performed for this review. This lack of consistent reporting quality may lead to the argument that “evidence-based art” is still far from acknowledged standards known from evidence-based medicine in general. Some authors even argue that artwork could not be an object of scientific research due to its immanent complexity [4]; however, if artists claim a positive effect of their work on patients and clinicians use art in hospitals as part of the healing environment, both have to accept that the effects of their actions have to be validated by scientific research like any other medical procedure [56].

Quality may be an independent confounder when investigating preferences for art styles. Comparing high quality examples of one art style with low-quality examples of another art style and concluding from superiority of the high-quality group that the high-quality art style would be superior to the low-quality art style could yield bias due to this quality difference. This was addressed in a study of Nanda et al. by using only highly requested popular art in all groups and pairing thematically similar pictures of the investigated art styles [13]*.*

Habituation may limit the effect duration of static pictures; however, some intrinsic properties of artwork may decelerate habituation and engender repeated attention or longer attention. Such properties seem to avoid habituation by eliciting strong positive or negative emotions or cognitive challenges. They may include provocative elements, intricate structures requiring longer scanning time, safe and serene contents, motives with valuable resources or repetitive biological-looking textures [46, 57]. In one study it was shown that the effect of viewing art works on mood may depend on the way of watching it. In this investigation of 97 participants a positive effect of art viewing on mood was significant for persons who were directed while regarding art works only. The participants were directed to think about the art they saw or paint or write themselves inspired by the art [58]*.*

## Conclusion

The results of this scoping literature analysis suggest that figurative and abstract art may both have positive effects on well-being and findings, albeit on a weak level of evidence. Due to the important role of nature scenes presented in photographs and paintings in most studies it seems reasonable to place natural views in patient and operating rooms that are acceptable for most patients. The opportunity to choose the artwork in their rooms may further enhance the effects on patient satisfaction, feeling of self-control and distraction by attractive stimuli. For social areas and public rooms a mixture of abstract and figurative art seems to be adequate that meets various interests, promotes social interaction and avoids mental deprivation by providing multiple sensual stimuli. The distractive properties of art could be beneficial during and after painful procedures or during chronic pain therapy.
